# The effectiveness of the food and beverage industry’s self-established uniform nutrition criteria at improving the healthfulness of food advertising viewed by Canadian children on television

**DOI:** 10.1186/s12966-018-0694-0

**Published:** 2018-06-22

**Authors:** Monique Potvin Kent, Jennifer R. Smith, Elise Pauzé, Mary L’Abbé

**Affiliations:** 1 School of Epidemiology and Public Health, Faculty of Medicine, University of Ottawa, 600 Peter Morand Cres., Room 301J, Ottawa, ON K1G5Z3 Canada; 2 Department of Nutritional Sciences, Faculty of Medicine, University of Toronto, Toronto, Canada

**Keywords:** Food and beverage marketing, Self-regulation, Nutrition criteria, Children, Television, Policy, Canada

## Abstract

**Background:**

Food and beverage marketing has been identified as an environmental determinant of childhood obesity. The purpose of this study is to assess whether the Uniform Nutrition Criteria established and implemented by companies participating in the self-regulatory Canadian Children’s Food and Beverage Advertising Initiative (CAI) had an impact on the healthfulness of food and beverage advertising during television programming with a high share of children in the viewing audience.

**Methods:**

Data on food advertising were licensed from Numeris for 27 television stations for Toronto for May 2013 and May 2016 (i.e. before and after the implementation of the nutrition criteria). First, television programs that had a child audience share of ≥35% (when the nutrition criteria applied) were identified. Ten percent of these programs were randomly selected and included in the study. After identifying the food and beverage ads that aired during these programs, the nutritional information of advertised products was collected and their healthfulness was assessed using the Pan-American Health Organization (PAHO) and UK Nutrient Profile Models (NPM). The healthfulness of CAI products advertised in May 2013 and 2016 was compared using Chi-square tests.

**Results:**

Although in May 2016, products advertised by CAI companies were more likely to be categorized as healthier by the UK NPM (21.5% versus 6.7%, χ^2^(1) = 12.1,*p* = 000) compared to those advertised in May 2013, the frequency of advertised products considered less healthy in May 2016 remained very high (78.5%) and comparable to that of products advertised by companies not participating in the CAI (80.0% categorized as less healthy). Furthermore, in both May 2013 and May 2016, 99–100% of CAI advertisements featured products deemed excessive in either fat (total, saturated, trans), sodium or free sugars according to the PAHO NPM.

**Conclusions:**

Despite modest improvements noted after the implementation of the CAI’s Uniform Nutrition Criteria, the healthfulness of most products advertised during programs with a high share of children in the viewing audience remains poor. Mandatory regulations are needed.

## Background

Food and beverage marketing has been identified as one factor driving the upward trend in global obesity rates among children [1, 2]. Indeed, an extensive body of research has shown that children’s exposure to this marketing, much of which promotes food and beverages of low nutritional quality, influences their dietary preferences, purchasing behaviors, and consumption patterns [1–4]. Based on this evidence, the World Health Organization has urged countries to develop policies to protect children from the marketing of unhealthy food and beverages [5].

In Canada, childhood obesity has tripled over the last three decades and currently more than 30% of children and youth have excess weight or obesity [6]. In the province of Quebec, commercial advertising to children has been banned since the 1980s. In all other provinces in Canada, food and beverage marketing to children is self-regulated by industry. In 2007, the Canadian Children’s Food and Beverage Advertising Initiative (CAI) was implemented by 16 food companies. Currently 18 companies participate including Coca Cola, Danone, General Mills, McDonald’s, and Nestlé, among others (see Table 1) [7]. Under this initiative, eleven companies have committed to not advertise to children less than 12 years old while the remainder have pledged to exclusively advertise “better-for-you” products (as defined by the companies themselves) in various media including television [8]. Each company established what constituted advertising to children, determined its own nutrition criteria defining which products are healthy enough to advertise to children, and set child audience thresholds that range from 25 to 35% (i.e. the percentage of the audience that must consist of children under 12 years of age before the pledges apply). For example, Hershey Canada has pledged to not advertise at all during television programs where children make-up 30% of the audience, while Kellogg’s has committed to only advertise “better-for-you” products, such as Froot Loops cereal, when children make-up 35% or more of the viewing audience [8].

**Table 1:** List of companies participating in the Canadian Children’s Food and Beverage Advertising Initiative by commitment

Companies pledging to not advertise to children under 12 years old in various media	Companies pledging to only advertise “better-for-you” products to children under 12 years old in various media
Coca-Cola Ltd. Ferrero Canada Ltd. Hershey Canada Inc. Kraft Canada Inc. Maple Leaf Food Inc. Mars Canada Inc. Mondelẽz Canada Nestlé Canada PepsiCo Canada ULC Unilever Canada Inc. Weston Bakeries Limited	Campbell Company of Canada Danone Inc. General Mills Canada Corporation Kellogg Canada Inc. McDonald’s Restaurants of Canada Limited Parmalat Canada Inc. Post Foods Canada Inc.

Since its implementation, the CAI has been criticized for low participation rates, high child audience thresholds, lax nutritional standards, and very narrow definitions of what constitutes advertising to children [9]. Research in Canada has concluded that the CAI is insufficiently protecting children from food and beverage marketing on television and the Internet [9–14]. Indeed, Canadian children (outside of Quebec) view on average between 4 and 7 food ads per hour per station [15, 16], and the majority of products advertised are unhealthy and high in sugar, fat and sodium [16]. Evaluations conducted before and after the implementation of the CAI have shown that these self-regulatory pledges are not limiting children’s exposure to food and beverage advertising on television. In fact, children’s exposure to this type of advertising increased between 2006 and 2009 [11] and the healthfulness of advertised products on children’s specialty channels did not improve [9].

In 2014, Uniform Nutrition Criteria were developed by participating CAI companies and these were fully implemented by December 2015 [7]. These criteria, based on 18 different nutritional recommendations, specify nutrition criteria for 8 product categories including: milk and alternatives, grains, soups, meat and alternatives, vegetables and fruit, occasional snacks, mixed dishes, and meals on the go. No nutrition criteria were established for chocolate, candy, and soft drinks because, as stated by the CAI, these foods would not be advertised to children under the age of 12. Nutrients to limit, as identified in the Uniform Nutrition Criteria, include calories, saturated fat, trans fat, sodium, and total sugars while nutrients to encourage include vitamin D, calcium, potassium, and fibre [7]. A total of 26 products are listed as compliant with the Uniform Nutrition Criteria and approved for advertising to children [8].

Though Advertising Standards Canada (ASC), the organization that administers the CAI, undertakes a yearly compliance review [8], no research to date has evaluated the impact of these new criteria using nutrient profile models used and accepted in the research community. The objective of this study was to fill this gap and assess whether the CAI Uniform Nutrition Criteria has improved the healthfulness of food/beverage advertising during television programming where children make-up a large share of the viewing audience. It was hypothesized that, after its implementation, the Uniform Nutrition Criteria would improve the healthfulness of the advertising seen by children during programming with a high share of children in the viewing audience. It was also hypothesized that the healthfulness of products advertised by CAI companies in May 2016 would be significantly better during television programs with a child audience share of at least 35%, where the new nutrition criteria applied, compared to television programs with a lower child audience share, where it did not.

## Methods

A quasi-experimental pre-post design with a control group was used in this study to compare the nutritional quality of foods/beverages advertised to children aged 2–11 when viewership of this age group was equal to or greater than 35% in May 2013 (before the development of the Uniform Nutrition Criteria) and in May 2016 (after its implementation). The control group consisted of the nutritional quality of food/beverage advertisements in May 2016 when child viewership ranged from 15 to 34.9%.

Television ratings data were obtained under license for 19 food categories from Numeris for May 2013 and May 2016 for 27 television stations (9 conventional and 18 speciality channels) for Toronto, the largest broadcast audience in Canada. These food categories (defined in Table 2) were selected as they are those that are the most advertised to children [9, 12, 17]. The month of May was selected as there are no holidays in this month that could potentially distort advertising expenditures.

**Table 2:** Definitions of Nielsen food categories that are included in the study

Food category	Description
Cakes	All cakes and puddings including items that are ready to eat or require additional preparation (excludes frozen pastry and pie shells)
Candy	Confectionary made form sugar, water, flavoring and food coloring (excludes candy with chocolate)
Cold cereals	Ready-to-eat products marketed as breakfast food (excludes infant cereals and oatmeal)
Cheese	Cheese products in various formats e.g. brick, string or slice (excludes cottage cheese)
Chocolate bars	Individually wrapped chocolate and candy bars (excludes boxed chocolate and candy with chocolate)
Compartment snacks and lunch kits	Prepackaged products comprised of two or more ingredients in separate compartments sold as portable snacks or meals
Cookies	Small baked sweet biscuits
Ice cream	Includes ice cream, frozen yogurt, sherbet, sorbet and frozen treats made from these foods
Pizza	Pizza not sold in restaurants
Portable snacks	Cereal, protein or fruit bars and squares, and fruit snacks
Fast food restaurants	Foods sold at restaurants where ordering is conducted at a counter or drive-through, where menu boards are place above the counter, and the table is cleaned up by the customer
Non fast food restaurants	Restaurant that serves prepared food and beverages that are ordered from a menu once seated and are consumed on the premises.
Snack foods	Savory snacks such as chips, pretzels, cheese puffs and meat-based snacks like jerky (excludes crackers)
Regular soft drinks	Any non-alcoholic carbonated drink
Diet soft drinks	Diet versions of soft drinks
Energy drinks	Drink products that are primarily consumed for the purpose of boosting one’s mental and physical stimulation
Sport drinks	Drink products that are meant to be consumed to rehydrate the body and replace electrolytes lost during physical activity
Juices, drinks and nectars	Sweetened and unsweetened juices and beverages that come in liquid, frozen, concentrated, and powdered forms (excludes water, milk and alternatives, tea and coffee drinks, cocktail mixers, and alcoholic beverage)
Yogurt	Yogurt in tub, tube and drink form (excludes frozen yogurt)

Using Nielsen Media Research Borealis ™ analytical software, it was determined which television programs had a child viewership of 15 to 34.9% and which had a viewership of ≥35%. The lower limit of 15% was chosen because it is the child viewership threshold applied in the province of Quebec, where all commercial advertising to children under the age of 13 has been legally prohibited since 1980 [18]. Children included those between the ages of 2 and 11 as the CAI guidelines apply to children under 12 years of age. The ***≥***35% level was selected as most CAI companies (*n* = 15) have a viewership threshold of 35% meaning that 35% of the audience must consist of children 2–11 years old before the CAI pledges apply. A total of 1536 television programs in May 2013 and 1289 in May 2016 met the ≥35% child viewership criteria while 1832 programs met the 15–34.9% viewership criteria for May 2016 (Table 3). For reasons pertaining to feasibility including time and resource constraints, only 10 % of these program samples were selected using a random number generator and were included in study. Using Nielsen Media Research Spotwatch™ software, the food/beverage ads that appeared during the first 30 min of each of these programs were identified.

**Table 3:** Number of television programs, sample selected and number of food/beverage advertisements in ***≥***35% and 15–34.9% child audience threshold samples for May 2013 and May 2016

	May 2013			May 2016		
	Television Programs *n* (% total programs)	Sample of Television Programs *n*	Food Ads in Sample *n*	Television Programs *n* (% total programs)	Sample of Television Programs *n*	Food Ads in Sample *n*
Programs reaching *≥*35% child audience threshold	1536 (6.2)	154	242	1289 (5.2)	129	334
Programs reaching 15–34.9% child audience threshold	1894 (7.6)	189	396	1832 (7.4)	183	416
All programs	24,960 (100)		81,056	24,910 (100)		87,121

Sources: Numeris and Nielsen Media Research, 2013, 2016

Each food advertisement was classified as a product ad (if a food and/or beverage were featured) or as a brand ad (if no specific product was featured). Each ad was also classified as to whether it belonged to a company participating in the CAI as of November 2016 (CAI) or not (non-CAI).

### Nutritional analysis

The nutrition information of products featured in each ad was collected. Nutrition data for the foods advertised in May 2013 was taken from the Food Label Information Program (FLIP) [19] which is a branded food composition database. FLIP data from 2013 contains information on ~ 15,500 products from the four largest national retailers by sales (Loblaws, Sobeys, Metro, Safeway), representing approximately 75% of the Canadian food retail market share. Nutrition information for products advertised in May 2013 not found in the FLIP database (essentially fast food and restaurant foods) and those advertised in May 2016 was collected, in order of priority, from Canadian company websites, the Nutrition Fact table on the product found in store, U.S. company websites, or the Canadian Nutrient File.

Collected information included: calories (kcal), total fat (g), saturated fat (g), trans fat (g), sodium (mg), carbohydrate (g), fibre (g), sugars (g), and protein (g) per stated serving size. The specific density (g/mL) of beverages was used to convert servings from millilitres to grams [20]. All nutrition information was then converted to 100 g servings.

The healthfulness of advertised foods and beverages was assessed using two nutrient profile models namely, the Pan American Health Organization Nutrient Profile Model (PAHO NPM) [21] and the UK Nutrient Profile Model (UK NPM) [22]. The former was selected as it considers only negative nutrients (e.g. sodium, free sugars, total fat, saturated fat, and trans fat) and classifies foods more stringently while the latter was selected as it considers both positive and negative nutrients and has been shown to classify foods less stringently and consistently with decisions made by dietitians [23]. The UK NPM has also shown to have good construct, convergent, and discriminate validity [24].

The PAHO NPM was used to classify advertised food/beverages according to whether they were excessive in total fat (≥30% of total energy from total fat), saturated fat (≥ 10% of total energy), trans fats (≥ 1% of total energy), sodium (≥ 1 mg per kcal) or free sugars (≥ 10% of total energy) [21]. Foods were also classified as excessive or not in at least one of these nutrients. The PAHO NPM was modified by applying it to all foods, including unprocessed foods, rather than applying it to processed or ultra-processed foods only. The free sugar content of foods was estimated using formulas suggested by the PAHO NPM [21].

The UK NPM, was also used to assess the healthfulness of advertised foods in May 2013 and May 2016 using the three-step process developed by the Food Standards Agency in the UK [22]. This model scores foods based on their content in energy, saturated fat, total sugar, sodium, fruit/vegetable/nut, fibre, and protein per 100 g serving. Foods that score 4 points or more and beverages that score 1 point or more are categorized as ‘less healthy’ [22]. Foods that do not fall into this category are defined as ‘healthier’.

When multiple food products were shown in the same advertisement, the ad was classified as excessive in fat, sodium and/or sugar as assessed by the PAHO NPM or as less healthy according the UK NPM if it featured at least one product that was categorized as such.

### Data analysis

Nielsen’s 19 food categories were condensed by grouping similar products to create 9 more meaningful categories. These included: cold cereal; candy and chocolate; cakes, cookies and ice cream; juice, soft drinks (regular and diet), sports drinks and energy drinks; pizza; compartment snack foods and portable snacks; restaurants (fast food and non-fast food); cheese; and yogurt. The frequency of ads by food categories and CAI participation were tabulated and the percentage change between May 2013 and May 2016 was calculated. Statistical tests (Mann-Whitney U test) compared the energy, total fat, saturated fat, trans fat, carbohydrates, sugar, protein, fibre, and sodium content per 100 g serving of foods and beverages featured in May 2013 advertisements to those in May 2016 for CAI and non-CAI companies. When ads featured multiple products, the nutrition information for the least healthy product as assessed by the UK NPM (i.e. the product with the highest score) was used for this analysis. If several products within the same ad tied for the highest score, one product was randomly selected using a random number generator. Chi-square tests were conducted to determine if the healthfulness of advertised foods as classified by the PAHO and UK NPMs during programming with a high child audience share changed between May 2013 and May 2016. The Mann-Whitney U and Chi-square tests described above were conducted for the ≥35% child viewership sample. Further comparisons were made between products advertised by CAI companies when child viewership was 15–34.9% and ≥ 35% in May 2016. The healthfulness of products advertised in May 2013 and May 2016 when child viewership was at least 35% was also compared by food category using Fisher Exact Tests.

## Results

### Product versus brand advertising

In May 2016, 0.5% (*n* = 2) and 2.1% (*n* = 7) of total ads were brand advertisements in the 15–34.9% and ≥ 35% child viewership samples, respectively. The remainder were products ads. There were no brand ads in the 35% viewership sample in May 2013.

### Frequency of food/beverage advertising per food category in May 2013 and in May 2016 (≥35% sample)

Overall, the frequency of food/beverage advertising was 38.0% higher in May 2016 compared to May 2013. The most frequently advertised product categories in May 2016 (as shown in Table 4) were restaurants (33.8% of total ads; 92.9% of which were fast food), candy and chocolate (18.0%), and cold cereal (15.3%). Among beverage categories advertised in May 2016 (*n* = 14), 81.3% were for juices, drinks, and nectars, 12.5% were for regular soft drinks, and 6.3% were for energy drinks (data not shown). In total, yogurt advertising was up by 217%, cold cereal advertising was up approximately 113%, while cheese advertising was up 81%, snack advertising was up 33%, and restaurant advertising was up 40% in May 2016 compared to May 2013. Advertising for fast food restaurants exclusively increased by 40.0% from May 2013 (*n* = 75) to May 2016 (*n* = 105) (data not shown).

**Table 4:** Frequency of food/beverage advertising in May 2013 and May 2016 per food category when child viewership reached at least ***≥***35%

	CAI^a^			Non-CAI^b^			Total		
	2013	2016		2013	2016		2013	2016	
Food Categories	*n* (%)	*n* (%)	Δ%	*n* (%)	*n* (%)	Δ%	*n* (%)	*n* (%)	Δ%
Cold cereal	24(20.0)	51(27.3)	112.5	0(0)	0(0)	–	24(9.9)	51(15.3)	112.5
Candy and chocolate	33(27.5)	31(16.6)	−6.1	24(19.7)	29(19.7)	20.8	57(23.6)	60(18.0)	5.3
Cakes, cookies and ice cream	8(6.7)	10(5.3)	25.0	4(3.3)	3(2.0)	−25.0	12(5.0)	13(3.9)	8.3
Juice, soft drinks, sports drinks and energy drinks	4(3.3)	6(3.2)	50.0	22(18.0)	10(6.8)	−54.5	26(10.7)	16(4.8)	−38.5
Pizza	0(0)	0(0)	–	0(0)	4(2.7)	–	0(0)	4(1.2)	–
Compartment snack foods and portable snacks	14(11.7)	16(8.6)	14.3	1(0.8)	4(2.7)	300.0	15(6.2)	20(6.0)	33.3
Restaurants (fast food and non-fast food)	26(21.7)	36(19.3)	38.5	55(45.1)	77(52.4)	40.0	81(33.5)	113(33.8)	39.5
Cheese	8(6.7)	18(9.6)	125.0	13(10.7)	20(13.6)	53.8	21(8.7)	38(11.4)	81.0
Yogurt	3(2.5)	19(10.2)	533.3	3(2.5)	0(0)	−100.0	6(2.5)	19(5.7)	216.7
Total	120(100)	187(100)	55.8	122(100)	147(100)	20.5	242(100)	334(100)	38.0

Source: Nielsen Media Research, 2013, 2016

^a^Companies participating in the Canadian Children’s Food and Beverage Advertising Initiative (CAI)

^b^Companies not participating in the CAI

Among CAI companies, the frequency of food/beverage advertisements was 55.8% higher in May 2016 compared to May 2013. The CAI product categories that were the most frequently advertised in May 2016 were cold cereals (27.3%), restaurants (19.3%; all of which were for fast food) and candy and chocolate (16.6%). The largest increases in CAI advertising between May 2013 and May 2016 were for yogurt (533%), cheese (125%), cold cereals (113%), and juice and soft drinks (50%). Restaurant advertising, comprised entirely of fast food advertisements, increased 38.5% in May 2016 compared to May 2013.

### Nutrient content per 100 g of foods/beverages advertised in May 2013 and May 2016 (≥35% sample)

Overall, products advertised in May 2016 when child viewership was at least 35% contained more sodium (U = 44,057, z = 2.41, *p* = .016, *r* = 0.10), trans fat (U = 45,950, z = 3.78, *p* = .000, *r* = 0.16), fibre (U = 44,953, z = 3.12, *p* = 002, *r* = 0.13), and protein (U = 46,308, z = 3.58, p = .000, *r* = 0.15) per 100 g serving compared to those advertised in May 2013 (Table 5). In May 2016, products advertised by CAI companies contained fewer calories (U = 8962, z = − 2.92, *p* = .004, *r* = − 0.17) and total fat (U = 9628, z = − 2.03, *p* = .042, *r* = − 0.12) per 100 g serving than in May 2013.

**Table 5:** Nutrient content per 100 g of foods/beverages advertised in May 2013 and May 2016 when child viewership reached at least ***≥***35%, Mann-Whitney U test

	CAI companies^a^					Non-CAI companies^b^					Total				
	2013 (*n* = 120)	2016 (*n* = 186)				2013 (*n* = 121)	2016 (*n* = 141)				2013 (*n* = 241)	2016 (*n* = 327)			
Nutrient	Mdn	Mdn	U	z	*P*	Mdn	Mdn	U	z	*P*	Mdn	Mdn	U	z	*P*
Calories (kcal)	393	370	8962	−2.92	.004	211	280	10,500	3.22	.001	319	300	39,865	0.24	.811
Total fat (g)	11.4	5.0	9628	−2.03	.042	1.8	9.7	10,070	2.55	.011	7.3	7.9	41,050	0.86	.392
Saturated Fat (g)	2.3	2.1	9963	−1.59	.112	0.5	2.6	10,033	2.52	.012	1.9	2.3	41,830	1.26	.207
Trans Fat (g)	0.00	0.00	12,287	1.71	.087	0.00	0.04	10,581	3.65	.000	0.0	0.0	45,950	3.78	.000
Sodium (mg)	389	403	11,729	0.76	.450	103	382	10,448	3.14	.002	256	389	44,057	2.41	.016
Carbohydrates(g)	62.0	56.8	10,106	−1.40	.162	14.6	21.7	10,027	2.45	.014	23.8	31.1	41,051	0.85	.394
Fibre (g)	0.0	0.7	12,048	1.27	.205	0.0	0.8	10,285	3.16	.002	0.0	0.8	44,953	3.12	.002
Sugar (g)	28.2	20.0	10,330	−1.10	.271	7.0	4.1	8181	−0.57	.567	10.7	12.6	40,036	0.33	.743
Protein (g)	4.5	6.7	11,742	0.77	.440	2.4	8.9	11,052	4.15	.000	3.8	6.7	46,308	3.58	.000

^a^Companies participating in the Canadian Children’s Food and Beverage Advertising Initiative (CAI)

^b^Companies not participating in the CAI

### Healthfulness of foods advertised in May 2013 and May 2016 (≥35% sample)

Overall in 2016, according to the PAHO criteria, 68.4% of advertisements featured foods/beverages that were excessive in free sugar, 59.8% were excessive in total fat, 59.5% were excessive in sodium, 50.3% were excessive in saturated fat, and 29.1% were excessive in trans fats as shown in Table 6. According to the PAHO criteria, 100% of food advertisements in 2016 featured products classified as excessive in at least one of these nutrients while according to the UK NPM, 79.1% of ads featured products that were classified as ‘less healthy’. In May 2016, it was 1.5 times more likely that food advertisements were deemed excessive in total fat (49.8% versus 59.8%, χ^2^(1) = 5.64,*p* = .018) compared to those that aired in May 2013. Advertisements in May 2016 were also 1.7 times more likely to be deemed excessive in trans fat (19.9% versus 29.1%, χ^2^(1) = 6.25,*p* = .012) and sodium (46.5% versus 59.5%, χ^2^(1) = 9.48,*p* = .002) compared to May 2013. Conversely, advertisements in May 2016 were 1.7 times less likely to feature food deemed less healthy by the UK NPM compared to May 2013 (86.7% versus 79.1%, χ^2^(1) = 5.48,*p* = .019).

**Table 6:** Healthfulness of foods/beverages advertised in May 2013 and May 2016 when child viewership reached at least ***≥***35% according to PAHO criteria and the UK Nutrient Profile Model

	CAI companies^a^				Non-CAI companies^b^				Total			
	2013	2016			2013	2016			2013	2016		
	*n* (%)	*n* (%)	χ^2^ (df)	OR^c^	*n* (%)	*n* (%)	χ^2^ (df)	OR^c^	*n* (%)	*n* (%)	χ^2^ (df)	OR^c^
Excessive in total fat	61 (50.8)	98 (52.7)	0.10(1)	1.08	59 (48.8)	97 (69.3)	11.4(1)*	2.37	120 (49.8)	195 (59.8)	5.64(1)*	1.50
Excessive in saturated fat	65(54.2)	96 (51.6)	0.19(1)	0.90	58 (47.9)	68 (48.6)	0.01(1)	1.03	123 (51.0)	164 (50.3)	0.03(1)	0.97
Excessive in trans fat	12 (10.0)	45 (24.2)	9.69(1)*	2.87	36 (29.8)	50 (35.7)	1.04(1)	1.31	48 (19.9)	95 (29.1)	6.25(1)*	1.65
Excessive in sodium	53 (44.2)	109 (58.6)	6.10(1)*	1.79	59 (48.8)	85 (60.7)	3.75(1)	1.62	112 (46.5)	194 (59.5)	9.48(1)*	1.69
Excessive in free sugars	100 (83.3)	156 (83.9)	0.02(1)	1.04	81 (66.9)	67 (47.9)	9.63(1)*	0.45	181 (75.1)	223 (68.4)	3.04(1)	0.72
Excessive in at least one nutrient	119 (99.2)	186 (100)	–	–	120 (99.2)	140 (100)	1.16(1)	–	239 (99.2)	326 (100)	–	–
Less healthy as per UK NPM	112 (93.3)	146 (78.5)	12.1(1)*	0.26	97 (80.2)	112 (80.0)	0.00(1)	0.99	209 (86.7)	258 (79.1)	5.48(1)*	0.58

^a^Companies participating in the Canadian Children’s Food and Beverage Advertising Initiative (CAI)

^b^Companies not participating in the CAI

^c^OR = Odds ratio; Reference group (1): 2013

*ρ-value < 0.05

Among CAI companies, it was 2.9 and 1.8 times more likely that advertisements airing in May 2016 featured a product classified as excessive in trans fat (10.0% versus 24.2%, χ^2^(1) = 9.69,p = .002) and sodium (44.2% versus 58.6%, χ^2^(1) = 6.10,*p* = .014), respectively, compared to those advertised in May 2013. In both time periods, 99–100% of CAI advertisements featured products that were classified as excessive in at least one nutrient according to the PAHO NPM however the frequency of advertisements featuring less healthy products as per the UK NPM was significantly lower in May 2016 compared to May 2013 (93.3% versus 78.5%, χ^2^(1) = 12.1,*p* = .000).

### Healthfulness of products advertised in May 2013 and May 2016 by food category (≥35% sample)

Cold cereals advertised in May 2016, all of which belonged to CAI companies, were more likely to be excessive in sodium compared to those advertised in May 2013 (98.0% versus 33.3%, p = .000) As for restaurants, foods advertised by CAI companies (i.e. McDonald’s) in May 2016 were less likely to be deemed less healthy (88.5% versus 42.9%, *p* = .010) compared to those advertised in May 2013. This was also true for the total sample of restaurant advertisements (81.3% versus 63.6%, p = .010) (data not shown).

### Nutrient content per 100 g of foods/beverages advertised by CAI companies in May 2016 (≥35% vs. 15–34.9% sample)

According to Mann-Whitney U tests, foods/beverages advertised by CAI companies contained more sugar (Mdn = 10.9 g and 20.0 g, U = 24,994, z = 2.62, *p* = .009, *r* = 0.13) and protein (Mdn = 5.0 g and 6.7 g, U = 24,212, z = 1.99, *p* = .047, *r* = 0.10) per 100 g serving when child viewership was 35% or higher compared to 15–34.5% (Table 7).

**Table 7:** Nutrient content per 100 g of foods/beverages advertised by CAI companies in May 2016 when child viewership ranged from 15 to 34.9% or reached at least ***≥***35%

CAI companies^a^ May 2016						
	15–34.9% (*n* = 234)	≥ 35.0% (*n* = 186)				
Nutrient	Mdn	Mdn	U	z	*r*	*p*-value
Calories (kcal)	293	370	23,669	1.55	0.08	.122
Total fat (g)	7.9	5.0	21,274	−0.40	−0.02	.692
Saturated fat (g)	2.3	2.1	21,625	−0.11	−0.01	.912
Trans fat (g)	0.0	0.0	21,770	0.01	0.00	.994
Sodium (mg)	389	403	23,221	1.18	0.06	.237
Carbohydrates (g)	31.7	56.8	24,939	2.57	0.13	.010
Fibre (g)	0.0	0.7	23,489	1.50	0.07	.134
Sugar (g)	10.9	20.0	24,994	2.62	0.13	.009
Protein (g)	5.0	6.7	24,212	1.99	0.10	.047

^a^Companies participating in the Canadian Children’s Food and Beverage Advertising Initiative (CAI)

### Healthfulness of foods advertised by CAI companies in May 2016 (≥35% versus 15–34.9% sample)

There were no statistically significant differences in the healthfulness of products advertised by CAI companies in May 2016 as per the PAHO and UK NPMs when child viewership was ≥35% and 15–34.9% (Table 8).

**Table 8:** Healthfulness of foods/beverages advertised by CAI companies in May 2016 when child viewership reached at least ***≥***35% or ranged from 15 to 34.9% according to PAHO criteria and the UK Nutrient Profile Model

	CAI companies^a^- May 2016				
	15–34.9%	≥ 35.0%			
	*n*(%)	*n*(%)	χ^2^ (df)	OR^b^	*p*-value
Excessive in total fat	140 (59.8)	98 (52.7)	2.15(1)	0.75	.142
Excessive in saturated fat	127 (54.3)	96 (51.6)	0.30(1)	0.90	.587
Excessive in trans fat	66 (28.2)	45 (24.2)	0.86(1)	0.81	.354
Excessive in sodium	125 (53.4)	109 (58.6)	1.13(1)	1.23	.288
Excessive in free sugar	179 (76.5)	156 (83.9)	3.49(1)	1.60	.062
Excessive in at least one nutrient	227 (97.0)	186 (100)	–	–	–
Less healthy as per UK NPM	191 (81.6)	146 (78.5)	0.64(1)	0.82	.424

^a^Companies participating in the Canadian Children’s Food and Beverage Advertising Initiative (CAI)

^b^OR = Odds ratio, reference group (1): 15–34.9% program sample

*ρ-value < 0.05

## Discussion

### Impact of the Uniform Nutrition Criteria

As hypothesised, when using the less stringent UK NPM, the products advertised during television programming with a high child audience share were marginally healthier in May 2016 (when the Uniform Nutrition Criteria applied) compared to those advertised in May 2013 (when it did not). Despite these modest improvements, more than 75% of all food advertisements featured products categorized as ‘less healthy’ and all of them featured products deemed excessive in either fat (total, saturated, trans), sodium or free sugars according to the more stringent PAHO NPM. When we exclusively examined CAI advertisements, results were similar and the overall healthfulness of products advertised in May 2016 was comparable to that of non-CAI companies to which the Uniform Nutrition Criteria did not apply. Though we attempted to compare the healthfulness of products advertised between May 2013 and May 2016 by food category, the sample size of many categories was too small to reliably test differences. Some results suggest that the healthfulness of some product categories advertised by CAI companies may have improved (e.g. fast food) while others suggest a worsening (e.g. cold cereals).

Our results also showed that contrary to what was hypothesized, foods advertised by CAI companies in May 2016 were not healthier according to both NPMs when child viewership was at least 35% compared to those advertised when child viewership was 15–34.9%. Together, these results suggest that the CAI’s Uniform Nutrition Criteria has not been particularly effective at improving the healthfulness of food/beverage advertising viewed by children aged 2 to 11 on television. This finding is consistent with previous research in Canada [9–11], the United States [25–27] and other countries [28] which has shown that self-regulation has not led to meaningful changes in the healthfulness of products advertised to children on broadcast television. Given this lack of effectiveness, many national and international organizations have called for the introduction of statutory regulations [5, 29]. Quebec’s Consumer Protection Act that prohibits commercial advertising to children under 13 years is often lauded as a model for other countries thinking of developing child advertising restrictions [30]. Indeed, research has shown this law is having some positive impact on children’s exposure to food and beverage advertising [12, 16]. For instance, some children in Quebec are exposed to fewer food/beverage advertisements on television and this advertising features fewer promotional techniques designed to appeal to children [12]. However, since the Consumer Protection Act was not specifically designed to restrict unhealthy food/beverage advertising, children in Quebec are still exposed to a large volume of food and beverage ads that target adolescents and adults and the healthfulness of advertised products are only marginally healthier than those advertised to children outside Quebec [16].

To effectively protect children from unhealthy food/beverage advertising, robust nutrition criteria defining which products can be advertised to them need to be adopted. Consideration also needs to be given to limiting children’s exposure to the promotion of brands that are largely associated with unhealthy foods (even if an ad features a healthy product), as the effect of advertising likely extends to other products of the same brand, regardless of their healthfulness. Indeed, research has shown that branding affects children’s food preferences and choices [31, 32]. An experimental study carried out by Boyland et al. [33], for example, found that the exposure to television advertisements featuring a healthier fast food meal led to the increased liking for fast food among children but did not result in healthier food choices made in a hypothetical situation. One way of limiting the promotion of brands associated with unhealthy products to children would be to only permit the advertising of brands whose entire product line meets the established nutrition criteria. Alternatively, it has also been suggested that food brands be classified as healthy or unhealthy based on the five most purchased products sold under that brand [34]. Though we documented a slight increase in brand advertising in May 2016 compared to May 2013, one may expect companies to increase such advertising if more stringent self-regulatory (or statutory) restrictions solely based on nutrient profiling were to be implemented (and adhered to).

Some of our results make one question whether the CAI companies are, in fact, complying with the Uniform Nutrition Criteria. To illustrate, in our May 2016 study sample when child viewership was at least 35%, 15 candy, 16 chocolate bar, and 1 soft drink ads belonging to CAI companies were identified despite their pledge to not advertise these products to children under the age of 12 when child audience thresholds were equal to or exceeded 35%. Some of these non-compliant ads aired on child and youth oriented channels such as YTV, Teletoon, and Much Music during programs that could be expected to appeal to children. For example, M&M’s candy and McDonald’s beverages (including a fruit smoothie, Coca Cola, and iced coffee) were advertised on May 9, 2016 during Just for Laughs Gags airing at 8 pm on YTV where the share of child viewers reached 37.3%. Four non-compliant advertisements (two for M&Ms., one for Skittles and one for McDonald’s beverages) also aired on May 14, 2016 during Mighty Hercules between 9 and 9:30 pm on Teletoon where children made up 42.1% of the audience. Advertisements belonging to seven companies that have pledged to abstain from advertising when child viewership reaches 25–35% were also identified in our sample. Six companies who pledged to only advertise ‘healthier’ foods  advertised products not specifically listed as compliant with the Uniform Nutrition Criteria. Since this study did not assess whether these unlisted products met these nutrition criteria, we cannot say whether the latter six companies are complying with their voluntary commitment. Since ad time is purchased based on projected audience estimates, companies would likely argue that they are complying with the CAI and could not have known that child audiences would be higher than projected. Though this may be true, companies could choose to purchase ad time based on stricter child audience thresholds (also known as “guardbanding”) to increase the likelihood of true compliance [35]. The examples of non-compliance cited above, whereby candy and sugar-sweetened beverages were advertised during children’s programming, also suggest at the very least that some companies are not complying with the spirit of the CAI. Our findings differ from those published by Advertising Standards Canada (ASC) [8]. ASC’s 2016 compliance report identified no instances of non-compliance during spot checks that examined 48 h of children’s television programs airing on three child-targeted channels (Teletoon, YTV, and Nickelodeon) during select time periods (e.g., YTV was checked from 6 am to 9 am on weekdays and 6 am-12 pm on Saturday) [8]. The instances of non-compliance identified in our study were either identified on channels different from those checked by the ASC (e.g. Much Music, CTV) or aired outside the time frames that were examined (e.g. on YTV, after 6 pm). This discrepancy highlights the inadequacy of current monitoring activities led by advertising standard agencies that are industry-funded. Independent monitoring is clearly needed to assess the impact of food/beverage advertising restrictions as well as company compliance.

In addition to non-compliance, the healthfulness of products advertised by the CAI may have only been modestly better in May 2016 compared to May 2013 as measured by UK and PAHO NPMs because the Uniform Nutrition Criteria themselves are not very stringent. For example, over a third (10) of the 26 products that are listed as compliant and approved for advertising to children by the CAI are sugar-sweetened breakfast cereals and include Froot Loops, Frosted Flakes, Alpha Bit Cereal, and Lucky Charms. Fruit flavored snacks such as Fruit by the Foot and Fruit Gushers, whose most predominant ingredient is sugar [36, 37], are also among the approved products. Given that many of these products are considered less healthy by the UK NPM (and would be by any other sound nutritional standards), it is not surprising that most CAI advertisements would still be considered unhealthy after the implementation of the Uniform Nutrition Criteria during programming on which it applies. For example, 7 of the 8 compliant CAI products advertised in May 2016 in our sample were deemed less healthy according to the UK NPM. Even if CAI companies were to adopt more stringent nutrition criteria, the voluntary nature of the initiative would still limit its effectiveness in improving the healthfulness of products advertised to children.

Though not related to the CAI or the Uniform Nutrition Criteria, it is interesting to note that our study identified four Red Bull advertisements (one in May 2016 and three in May 2013) during programs where child viewership reached 35% even though Health Canada regulations prohibit the advertising of energy drinks to children [38]. Similar results have been found on 2 of 10 Canadian child preferred websites where ads for Red Bull appeared on websites where children aged 2–11 constituted more than 45% of website visitors [39]. The promotion of energy drinks to children is worrisome given the adverse health effects associated with their consumption including anxiety, sleep disturbances, cardiovascular and gastro-intestinal symptoms, and even seizures and death in some rare cases [40, 41]. Interestingly, Red Bull GmbH is a member of the Canadian Beverage Association, an industry interest group that claims that all its members “voluntarily commit to not advertise energy drinks in programming … whose primary target audience is children” (i.e. when children under 12 years constitute more than 35% of the audience) [42, 43]. The energy drink ads found in our sample are further evidence that voluntary pledges made by industry are ineffective in protecting children.

This study also found that the frequency of food/beverage advertisements was higher in May 2016 compared to May 2013 during programs where children made-up 35% or more of the viewing audience. During this programming, there were on average of 1.6 food/beverage ads per 30-min program in May 2013 while in May 2016, there were 2.6 ads per program. The frequency of ads belonging to CAI companies was also higher in May 2016 (1.4 ads/program) compared to May 2013 (0.8 ads/program). The increase in frequency may be due to a rise in total advertising during television programs though what remains clear is that children’s potential exposure to food/beverage advertising on television has increased.

### Strengths and limitations

This study is the first to evaluate the CAI Uniform Nutrition Criteria. Its strengths include the use of Numeris and Nielsen Media Research data and analytical software. Further, this study also applied two nutrient profile models, the PAHO and UK NPMs, which provided a comprehensive assessment of the healthfulness of products advertised to children. Though the UK NPM offered good reliability and validity, it is currently being reviewed to more accurately reflect the latest dietary guidelines, particularly as it pertains to sugar [44]. The use of the 2013 FLIP data was also a strength given that it coincided with the May 2013 advertising data. However, it did not include nutritional information for fast food therefore this data had to be drawn from 2016 data. Any fast food reformulation between 2013 and 2016 would therefore not be accounted for in our data. This research was also based on the advertising of 19 food categories frequently advertised to children on Canadian television stations. Therefore, our findings cannot be generalized to other food categories, other media, or to non-Canadian television stations. A final limitation is that our research does not specifically evaluate individual company compliance; it is therefore difficult to determine whether the Uniform Nutrition Criteria are to blame for the poor nutritional quality of food advertising to children or whether it is a question of companies not complying with the criteria (or both).

## Conclusion

This study adds to the body of evidence showing that industry self-regulation does not lead to substantive improvements in food/beverage advertising directed at children on television, further emphasizing the need for statutory restrictions. To protect children, food/beverage restrictions based on stringent nutrition criteria need to be adopted. The instances of non-compliance cited in this study also highlight the need for effective third-party monitoring to hold food and beverage companies accountable.

## Abbreviations

ASC: Advertising Standards Canada
CAI: Canadian Children’s Food and Beverage Advertising Initiative
FLIP: Food label information program
PAHO NPM: Pan American Health Organization Nutrient Profile Model
UK NPM: United Kingdom Nutrient Profile Model
